# Characterization of glucose uptake metabolism in visceral fat by ^18 ^F-FDG PET/CT reflects inflammatory status in metabolic syndrome

**DOI:** 10.1371/journal.pone.0228602

**Published:** 2020-02-06

**Authors:** Kisoo Pahk, Eung Ju Kim, Yong-Jik Lee, Sungeun Kim, Hong Seog Seo

**Affiliations:** 1 Department of Nuclear Medicine, Korea University Anam Hospital, Seoul, Republic of Korea; 2 Cardiovascular Center, Korea University Guro Hospital, Seoul, Republic of Korea; Weill Cornell Medical College in Qatar, QATAR

## Abstract

**Objective:**

The inflammatory activity of visceral adipose tissue (VAT) is elevated in metabolic syndrome (MS), and associated with vulnerability to atherosclerosis. Inflammation can be assessed by glucose uptake in atherosclerotic plaques. We investigated whether the glucose uptake of VAT, assessed by ^18^F-fluorodeoxyglucose positron emission tomography/computed tomography (^18^F-FDG PET/CT), is associated with systemic inflammatory status, and related to the number of MS components.

**Methods:**

^18^F-FDG PET/CT was performed in a total of 203 participants: 59 without MS component; M(0), 92 with one or two MS components; M(1–2), and 52 with MS. Glucose uptake in VAT was evaluated using the mean standardized uptake value (SUVmean) and the maximum SUV (SUVmax). Glucose uptakes of immune-related organs such as the spleen and bone marrow (BM) were evaluated using the SUVmax.

**Results:**

VAT SUVmax correlated with high-sensitivity C-reactive protein (hsCRP) and the SUVmax of spleen and BM, which reflect the status of systemic inflammation. Both hsCRP and the SUVmax of the spleen and BM were higher in the MS group than in the M(1–2) or M(0) groups. In VAT, SUVmax increased with increasing number of MS components, while SUVmean decreased.

**Conclusions:**

The SUVmax and SUVmean of VAT assessed by ^18^F-FDG PET/CT reflected inflammation-driven unique glucose metabolism in the VAT of MS patients, distinct from that of atherosclerotic plaques.

## Introduction

Obesity, especially visceral obesity, is associated with insulin resistance, hypertension, and dyslipidemia. Together, these symptoms constitute metabolic syndrome (MS) [1], which increases the risk of cardiovascular disease (CVD) [1]. The key pathophysiological mechanism underlying CVD risk, which is also associated with MS, is related to dysfunctional visceral adipose tissue (VAT) that facilitates chronic inflammation in atherosclerotic arterial lesions [2, 3].

It is well known that visceral obesity promotes a shift in VAT from a healthy to dysfunctional and inflammatory state [2–4]. In VAT dysfunction, VAT is activated and secretes pro-inflammatory cytokines, such as interleukin-6 (IL-6), tumor necrosis factor-alpha (TNF-α), and monocyte chemotactic protein-1 (MCP-1), thereby promoting infiltration of inflammatory cells, particularly macrophages [2–4], which further exacerbate the inflammatory state. Inflammation in VAT induces insulin resistance and systemic inflammation, which eventually contributes to an increased risk of CVD [2–4].

Glucose metabolism plays an important role in inflammation; immune cells increase glucose uptake to synthesize inflammatory bio-substrates [5]. The basic physiology of glucose metabolism during the inflammatory response is the underlying principle in ^18^F-fluorodeoxyglucose positron emission tomography/computed tomography (^18^F-FDG PET/CT), which is used to detect inflammation and its associated diseases [6, 7]. Despite the importance of VAT inflammation in CVD risk, current clinical methods for measuring inflammatory activity in the VAT of patients with MS are limited. In several recent studies, ^18^F-FDG PET/CT was performed to measure glucose uptake in VAT in obese populations [8–10]. It was reported that the mean standardized uptake value (SUVmean) of VAT was higher than that of subcutaneous adipose tissue (SAT). As VAT has a larger number of immune cells compared to SAT [8, 11], these cells may contribute to diffrential glucose metabolsim in VAT and SAT [8]. However, the SUVmean of VAT was unexpectedly lower in obese participants than in metabolically healthy, lean participants [9, 10]. Thus, the use of the SUVmean of VAT as an appropriate surrogate marker of inflammatory activity in VAT of patients with MS requires validation.

Histologically, adipose tissue consists of various cellular components, including adipocytes, immune cells, vascular tissue, and connective tissue matrix [11]. Of these, adipocytes and immune cells are primarily involved in VAT inflammation [12]. Adipocytes are not typically involved in inflammation. However, in obesity, enlarged adipocytes become dysfunctional and promote inflammation through the secretion of TNF-α, IL-6, and IL-1β [12, 13]. Metabolically, in obesity-related VAT inflammation, adipocytes become insulin-resistant due to the reduced expression of insulin-dependent glucose transporter-4 (GLUT-4), and display decreased glucose uptake, despite being involved in inflammation [14, 15]. However, inflammatory cells dispersed among dysfunctional adipocytes do not develop insulin resistance, because these cells utilize glucose for the synthesis of inflammatory bio-substrates, mainly through insulin-independent GLUT-1 glucose transporters [16, 17]. Therefore, because insulin resistance occurs exclusively in adipocytes, and not in immune cells in inflamed adipose tissues, the glucose uptake metabolism of adipocytes could be expected to gradually differ from that of immune cells with increasing degrees of inflammation in VAT. However, little research has been conducted on this pathophysiological feature during VAT inflammation in humans.

We hypothesized that: i) the SUVmean reflects the average glucose uptake by adipocytes composed mostly of VAT and is partially affected by those of scattered immune cells in VAT; ii) the maximum standardized uptake value (SUVmax) of ^18^F-FDG better reflects the glucose uptake of immune cells in VAT, and iii) in MS patients, inflammatory activity in VAT will be increased, leading to an increased SUVmax and decreased SUVmean.

In this prospective ^18^F-FDG PET/CT study of VAT, we investigated whether the SUVmax of VAT, is correlated with the index of systemic inflammation and whether SUVmax changes according to the number of MS components, compared to the SUVmean.

## Methods

### Participants and design

**Fig 1:**
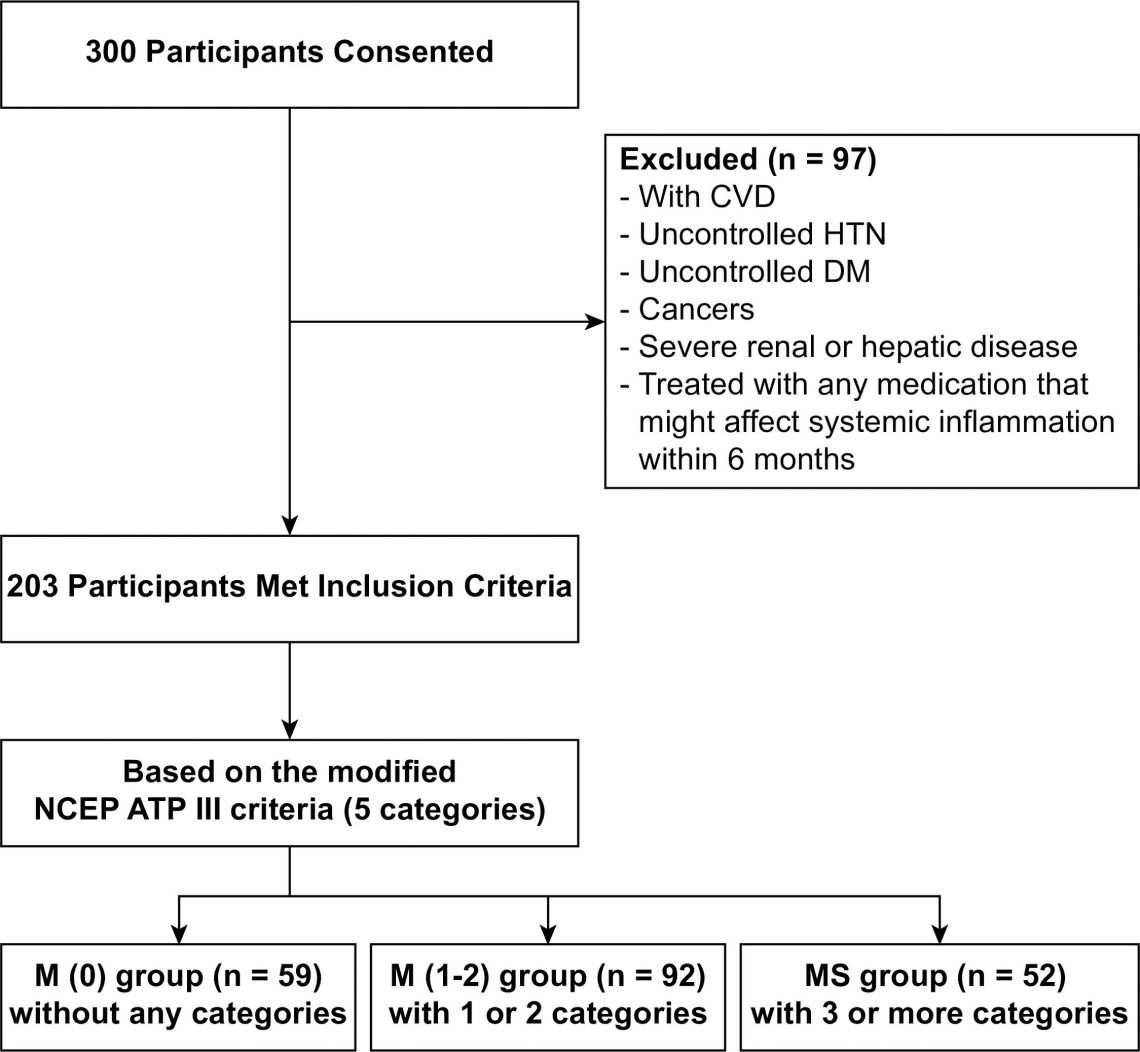
Flow chart of participant selection. CVD, cardiovascular disease; HTN, hypertension; DM, diabetes mellitus; NCEP ATP III, National Cholesterol Education Program Adult Treatment Panel III.

A total of 300 participants were prospectively recruited at the Korea University Guro Hospital between June 2008 and March 2009. MS was diagnosed when three or more of the following five categories from the modified NCEP ATP III (National Cholesterol Education Program Adult Treatment Panel III) in Korea were present: 1) abdominal obesity: waist circumference ≥90 cm in men and ≥80 cm in women; 2) fasting triglycerides ≥150 mg/dl; 3) high-density lipoprotein (HDL) cholesterol <40 mg/dl in men and <50 mg/dl in women; 4) systolic blood pressure ≥130 mmHg or diastolic blood pressure ≥85 mmHg (either value) or use of antihypertensive medication; and 5) fasting plasma glucose ≥100 mg/dl or use of antidiabetic medication [1, 18]. Participants with CVD, uncontrolled hypertension, uncontrolled diabetes mellitus (DM), cancers, severe renal or hepatic disease, or participants treated with any medication that might affect systemic inflammation within six months of the present study were excluded. Study participants were divided into three main groups: the M(0) group, comprised of participants without any of the five categories of modified NCEP ATP III in Korea; the M(1–2) group, comprised of participants with one or two categories; and the MS group, comprised of participants with three or more categories. Finally, a total of 203 participants (102 males and 101 females) were enrolled and underwent ^18^F-FDG PET/CT (Fig 1). The study conformed to the guidelines of the Declaration of Helsinki and was approved by the institutional review board at Korea University Guro Hospital (approval no. KUGH13015) and all participants provided written informed consent. Patents records/information was anonymized and de-identified prior to analysis.

### Medical history, and anthropometric and biochemical measurements

We evaluated the use of medication and current smoking habits through interviews and medical records from the Korea University Guro Hospital. Body mass index (BMI) was calculated as weight/height squared (kg/m^2^). Waist circumference (WC) was measured in a standing position at the level of the umbilicus.

All blood samples were acquired after overnight fasting. Levels of lipids, including total cholesterol, triglyceride, high-density lipoprotein cholesterol, and low-density lipoprotein cholesterol were measured using a chemistry analyzer (Hitachi 747, Hitachi, Tokyo, Japan). The fasting glucose level was measured using a compact glucometer (Accu-Check, Roche Diagnostics, Indianapolis, IN, USA) prior to FDG injection. The levels of high-sensitivity C-reactive protein (hsCRP) levels were measured by using Dade Behring BNII analyzer (Siemens, Munich, Germany).

### ^18^F-FDG PET/CT protocol

All participants fasted for at least 6 h before undergoing ^18^F-FDG PET/CT to maintain a blood glucose level of <180 mg/dL. The PET/CT scan was started 1 h after injection of 5.29 MBq/kg (0.14 mCi/kg) ^18^F-FDG using an integrated PET/CT scanner (GEMINI TF, Philips Medical Systems, Cleveland, OH, USA), which is a time-of-flight capable and fully three-dimensional scanner composed of a lutetium-yttrium oxyorthosilicate full-ring PET scanner and 16-slice helical CT scanner. The whole-body PET/CT scan covered the region from the skull-base to the proximal thigh. The CT scan (120 kVp, 50 mA, 4 mm slice thickness) was performed without contrast material at first, then followed by the PET scan. The PET unit had an 18 cm axial field of view with a 4.4 mm spatial resolution and the scan was performed for 9 bed positions at 1 min per bed position. CT images were reconstructed on a 512 × 512 matrix and later converted into 511 keV-equivalent attenuation factors for the attenuation correction of the data. The PET images were reconstructed on a 128 × 128 matrix using an iterative algorithm (three-dimensional row-action maximum likelihood algorithm).

### Image analysis

Images were reviewed by two experienced nuclear medicine physicians (KP and SK) using a dedicated commercially available workstation (Extended Brilliance Workspace version 3.5, Philips Healthcare, Eindhoven, Netherlands). The physicians were blinded to participants’ clinical information.

VAT and SAT were identified in CT images based on predefined Hounsfield units (ranging from -70 to -110), as previously described [8, 19–21]. VAT was defined as the adipose tissue in the intra-abdominal fat region and SAT was defined as the adipose tissue in the extra-peritoneal fat region, between the skin and muscle. Next, glucose uptake of the adipose tissue was quantified by drawing a region of interest (ROI) around each adipose tissue on a CT slice, which led to the automatic generation of the same ROIs on the transaxial PET images. Standard, circular ROIs were placed on the VAT and SAT regions, and SUV was calculated as follows:

SUV=Traceractivity(ROI)(MBq/mL)/Injecteddose(MBq)/Totalbodyweight(g)

To determine the glucose uptake of the VAT, ROIs were selected on three consecutive slices of retroperitoneal VAT area, between the fourth and fifth lumbar vertebrae, and manually adjusted to exclude overspill ^18^F-FDG uptake in the vessel, intestine, and/or muscle, as previously described [9, 19–21]. The highest SUVs and mean SUVs from the three consecutive ROIs were recorded and the averages defined as the SUVmax and SUVmean of the VAT, respectively. For the evaluation of glucose uptake in SAT, three consecutive ROIs were drawn on the subcutaneous anterior abdominal wall, subcutaneous posterior back region, or the buttock area. The highest SUVs and mean SUVs from these three consecutive ROIs were acquired and the averages were determined as the SUVmax and SUVmean of the SAT, respectively. The results of intra-class correlation coefficient (ICC) showed good reproducibility for measurement of glucose uptake in fat regions between inter- and intra-observer (Table 1).

**Table 1:** Intra-class correlation coefficient (ICC) analysis for measurement of glucose uptake in fat regions between inter- and intra-observer.

	Inter-observer reliability		Intra-observer reliability	
	ICC	95% CI	ICC	95% CI
VAT SUVmax	0.963	0.951–0.972	0.989	0.985–0.992
VAT SUVmean	0.828	0.774–0.87	0.86	0.815–0.894
SAT SUVmax	0.918	0.892–0.938	0.93	0.908–0.947
SAT SUVmean	0.883	0.846–0.911	0.962	0.95–0.971

VAT, visceral adipose tissue; SAT, subcutaneous adipose tissue; SUVmax, maximum standardized uptake value; SUVmean, mean standardized uptake value; 95% CI, 95% confidence interval.

To assess the glucose uptake of the spleen and BM, the SUVmax of each was measured as previously described [7]. Briefly, ROIs were placed on the spleen and the third to fifth lumbar vertebrae based on the anatomical CT images. The highest SUVs from all ROIs on all transaxial slices were acquired and the average was used as the representative SUVmax for the entire organ. The glucose uptake of liver was also measured as like spleen.

### Statistical analysis

All data are presented as mean ± standard deviation. The Pearson chi-squared (χ^2^) test or Fisher’s exact test, and one-way analysis of variance (ANOVA) with post-hoc Tukey test were performed for comparison of multiple groups. Pearson’s correlation coefficient was used to evaluate the degree of correlation. To determine the factors associated with the SUV parameters of the VAT, multiple linear regression analysis was also performed. SPSS version 17.0 (SPSS Inc., Chicago, IL, USA) and MedCalc version 18.5 (MedCalc, Mariakerke, Belgium) was used for data analysis. A *p*-value of ≤ 0.05 was considered statistically significant.

## Results

### Systemic inflammation is increased in MS and is correlated with the number of MS components in VAT

Of the 203 participants, 52 were in the MS group, 92 were in the M(1–2) group, and 59 were in the M(0) group. The baseline characteristics of all participants are presented in Table 2.

**Table 2:** Baseline characteristics of study participants.

	M(0), n = 59	M(1–2), n = 92	MS, n = 52	*p*
Age, y	57.5 ± 9.2	55.8 ± 10.9	58 ± 10.8	0.31
Men, n (%)	31 (52.5)	50 (54.3)	21 (40.4)	0.204
BMI, kg/m^2^	23.2 ± 1.7	25.5 ± 3.2*	28.2 ± 4.1^†^^‡^	<0.001
WC, cm	79 ± 6.6	87 ± 7.7*	92.8 ± 8.7^†^^‡^	<0.001
Glucose, mg/dL	111.8 ± 34.9	112.1 ± 30	115.8 ± 27.6	0.198
Total cholesterol, mg/dL	179.1 ± 41.6	179.7 ± 46.9	179.4 ± 42.5	0.997
Triglycerides, mg/dL	115.2 ± 71	142.5 ± 143.6	246.2 ± 154.3^†^^‡^	<0.001
HDL cholesterol, mg/dl	53.2 ± 9.9	52.1 ± 12	44.1 ± 9.8^‡^	0.0006
LDL cholesterol, mg/dL	120.5 ± 38.5	112.2 ± 40	114 ± 35.6	0.764
Current smokers, n (%)	2 (3.4)	16 (17.4)*	8 (15.4)^†^	0.035
hsCRP, mg/L	0.75 ± 0.56	1.57 ± 1.81	3.25 ± 3.16^†^^‡^	<0.001
Liver SUVmax Medication, n (%)	2.72 ± 0.55	2.65 ± 0.55	2.98 ± 0.63^†^^‡^	0.005
Hypertension	0	50 (54.3)	42 (80.8)	0.002
Diabetes mellitus	0	14 (15.2)	22 (42.3)	<0.001
Lipid lowering	0	41 (44.6)	23 (44.2)	0.969

All data were presented as mean ± standard deviation or n (%). *P*-values were determined using ANOVA with post-hoc Tukey test for continuous variables and Pearson chi-squared (χ^2^) test or Fisher exact test for categorical variables.

*p ≤ 0.05, M(0) vs. M(1–2)

^†^*p* ≤ 0.05, M(0) vs. MS

^‡^*p* ≤ 0.05, M(1–2) vs. MS. MS, Metabolic syndrome; M(0), no MS components; M(1–2), one or two MS components; BMI, body mass index; WC, waist circumference; HDL, high-density lipoprotein; LDL, low-density lipoprotein; hsCRP, high-sensitivity C-reactive protein; SUVmax, maximum standardized uptake value.

**Fig 2:**
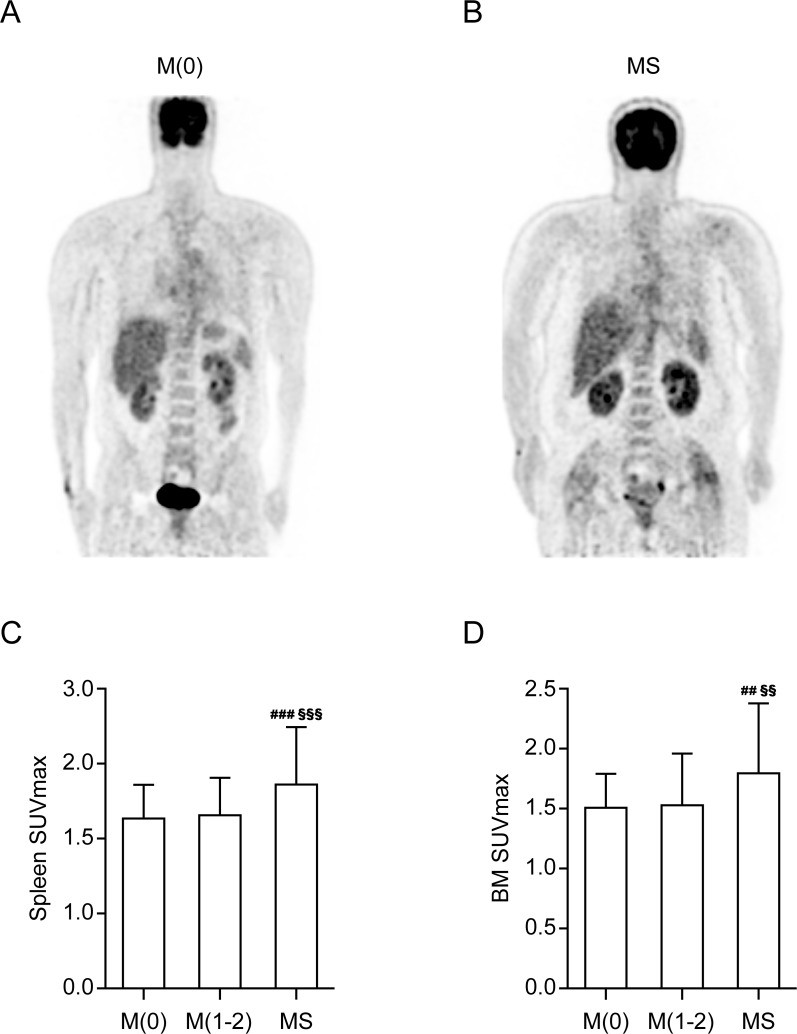
Representative examples of maximum intensity projection ^18^F-fluorodeoxyglucose (FDG) positron emission tomography/computed tomography (PET/CT) images showing increased bone marrow (BM) and spleen uptakes in (B) metabolic syndrome (MS) patient compared to (A) M(0) participants. A comparison of the maximum standardized uptake value (SUVmax) of the spleen (C) and the SUVmax of the BM (D) according to the number of MS components. M(0), *n* = 59; M(1–2), *n* = 92; MS, *n* = 52. M(0), no MS components; M(1–2), participants with one or two MS components; MS, three of more MS components. *P*-values were determined using ANOVA with post-hoc Tukey test. ^##^*p* ≤ 0.01; M(0) vs. MS, ^###^*p* ≤ 0.001; M(0) vs. MS. ^§§^*p* ≤ 0.01; M(1–2) vs. MS, ^§§§^*p* ≤ 0.001; M(1–2) vs. MS.

hsCRP and the SUVmax of the spleen and bone marrow (BM), well-known indices for systemic inflammation [7, 22], were significantly correlated with the number of MS components (Table 3) and were significantly increased in the MS group (Table 2 and Fig 2). Furthermore, hsCRP and the SUVmax of both the spleen and the BM were significantly correlated with the SUVmax of VAT (Table 4). In contrast, hsCRP and the both SUVmax of the spleen and the BM were not correlated with the SUVmean of VAT and the any SUV parameters of SAT.

**Table 3:** Correlation analysis between the hsCRP, the SUVmax of spleen and BM, and the number of MS components.

	Systemic inflammation markers		
	hsCRP	Spleen SUVmax	BM SUVmax
The number of MS components	0.393^†^	0.198*	0.203*

Correlation coefficients and *p*-values were calculated using the Pearson’s correlation analysis.

*p ≤ 0.01

^†^*p* ≤ 0.001. hsCRP, high-sensitivity C-reactive protein; MS, metabolic syndrome; SUVmax, maximum standardized uptake value; BM, bone marrow.

**Table 4:** Correlation analysis between metabolic parameters of adipose tissues and the hsCRP, the SUVmax of spleen and BM, and the number of MS components.

Parameters	VAT		SAT	
	SUVmax	SUVmean	SUVmax	SUVmean
hsCRP	0.25^†^	–	–	–
Spleen SUVmax	0.29^‡^	–	–	–
BM SUVmax	0.34^‡^	–	–	–
The number of MS components	0.17*	-0.18^†^	-0.17*	-0.17*

Correlation coefficients and *p*-values were calculated using the Pearson’s correlation analysis.

*p ≤ 0.05

^†^*p* ≤ 0.01

^‡^*p* ≤ 0.001.–not significant. hsCRP, high-sensitivity C-reactive protein; MS, metabolic syndrome; VAT, visceral adipose tissue; SAT, subcutaneous adipose tissue; SUVmax, maximum standardized uptake value; SUVmean, mean standardized uptake value; BM, bone marrow.

### Characteristics of ^18^F-FDG uptake in VAT and SAT, and correlation of SUV parameters of VAT and SAT with the number of MS components

**Fig 3:**
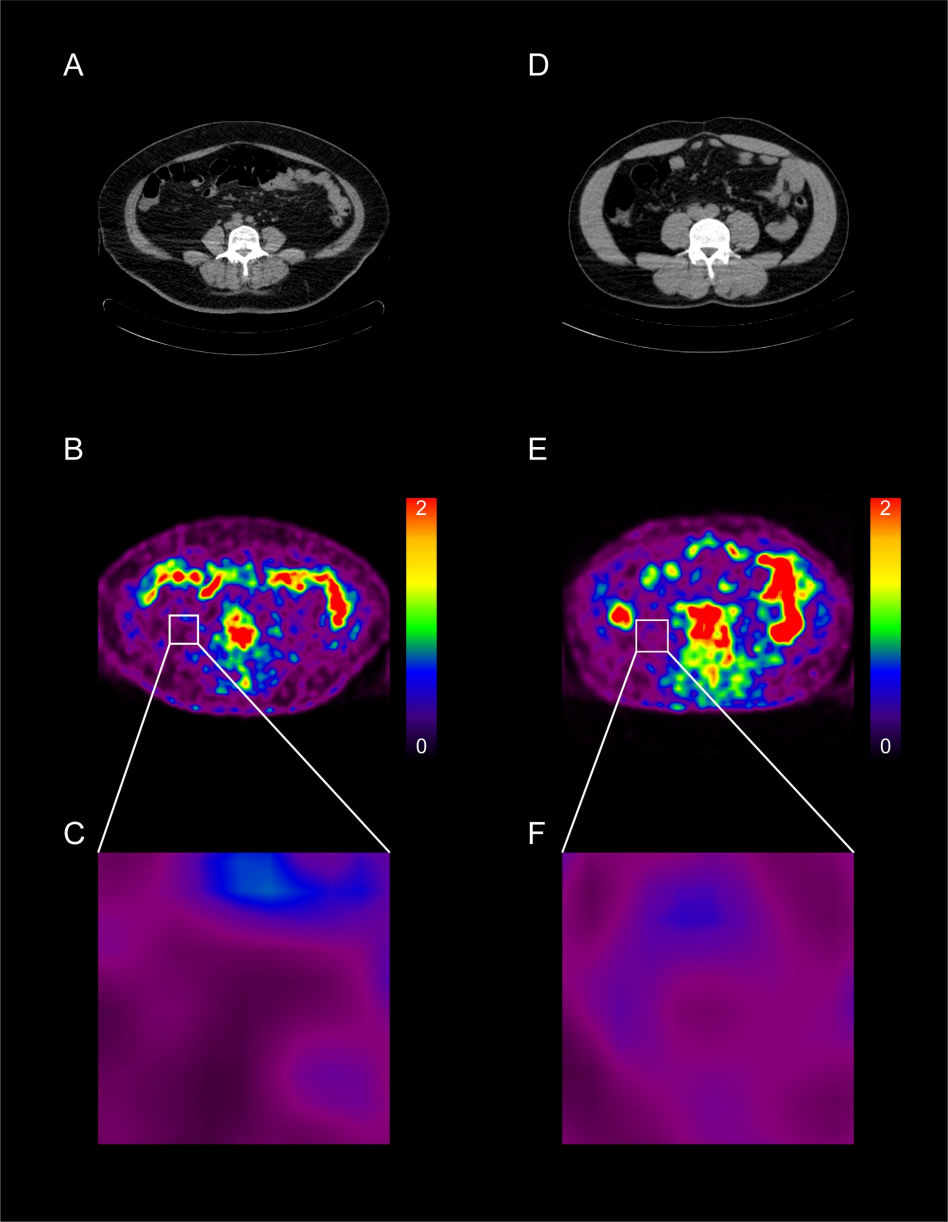
Representative images of ^18^F-FDG PET/CT analysis of VAT and SAT of a MS patient (A-C) and a M(0) patient (D-F). Transaxial CT images at the level of the L4 vertebrae were acquired in both MS and M(0) patients (A and D). Transaxial ^18^F-FDG images corresponding to the above transaxial CT images were acquired (B and E). Magnified ^18^F-FDG images of VAT in both MS and M(0) patients (C and F). SUVs are displayed in a color scale indicating high (red) to low (black) FDG uptake. Different FDG uptake pattern of the VAT region is observed between the MS and the M(0) patients. In the VAT region, MS patient exhibits a large portion of relatively lower FDG uptake area and a small portion of relatively higher FDG uptake area than those of M(0) patient.

**Fig 4:**
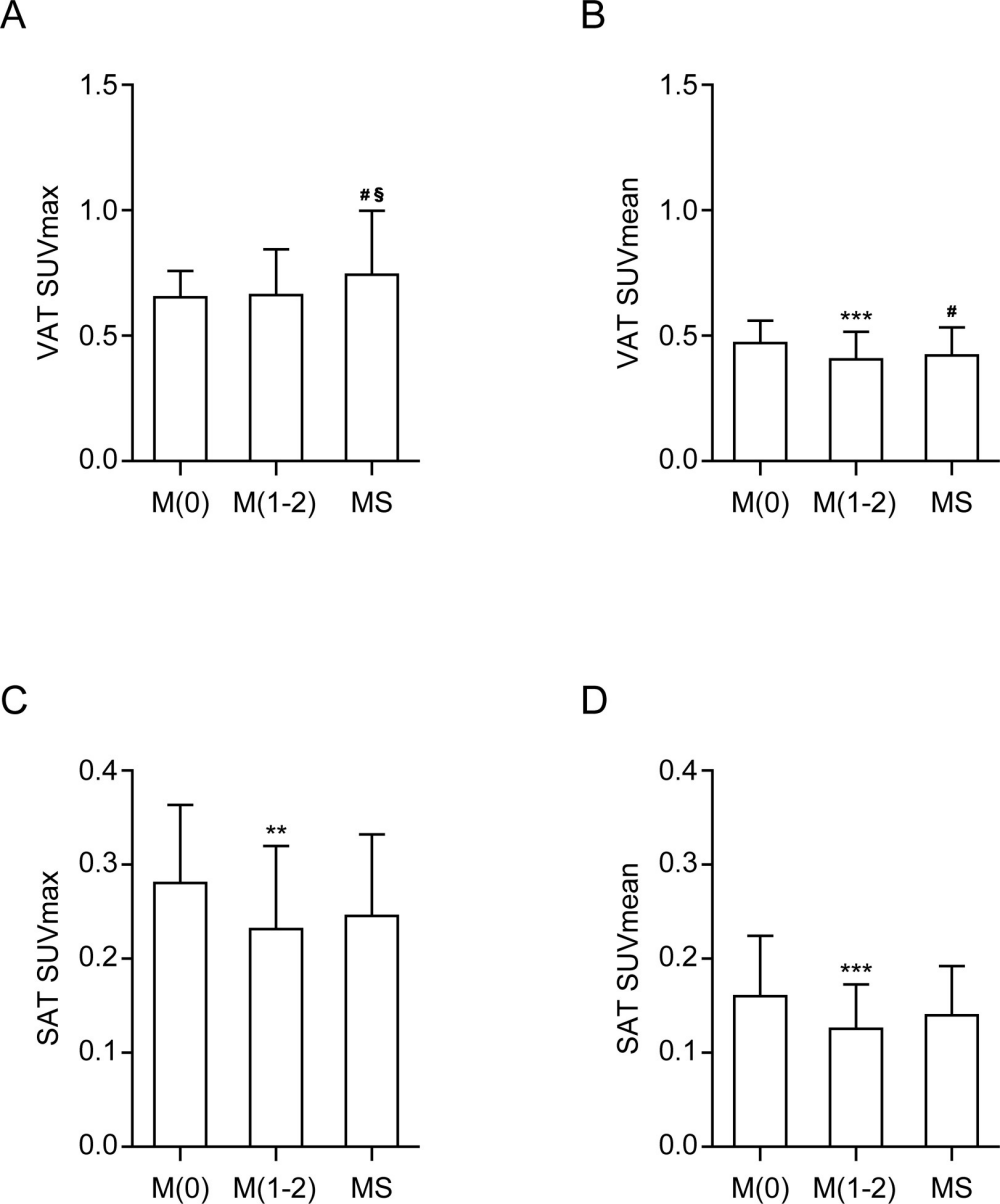
Comparison of (A) VAT SUVmax, (B) VAT SUVmean, (C) SAT SUVmax, and (D) SAT SUVmean according to the number of MS components. M(0), *n* = 59; M(1–2), *n* = 92; MS, *n* = 52. *P*-values were determined using ANOVA with post-hoc Tukey test. ^#^*p* ≤ 0.05; M(0) vs. MS. ^§^*p* ≤ 0.05; M(1–2) vs. MS. **p ≤ 0.01; M(0) vs. M(1–2), ***p ≤ 0.001; M(0) vs. M(1–2).

The SUVmax of VAT in the MS group was significantly higher than that in M(0) and M(1–2) groups (0.74 ± 0.25 vs. 0.65 ± 0.11 and 0.66 ± 0.18, respectively. *p* = 0.01. Figs 3 and 4A). However, the increase in the number of MS components did not show a gradual linear increase in the SUVmax of the VAT. Conversely, the SUVmean of VAT in both MS and M(1–2) groups were lower than those in the M0 group (0.42 ± 0.11 vs. 0.47 ± 0.09, *p* = 0.037, and 0.41 ± 0.11 vs. 0.47 ± 0.09, *p* = 0.001, respectively. Fig 4B). No significant difference between the SUVmean of VAT in the MS and M(1–2) groups was observed. Together these finding indicate that the SUVmax of VAT was positively correlated with the number of MS components, whereas the SUVmean was inversely correlated (Table 4).

The SUVmax of SAT in the MS group was lower than that in the M(0) group but not statistically significant (0.25 ± 0.09 vs. 0.28 ± 0.08, *p* = 0.088, Fig 4C) and was not different from that in the M(1–2) group (0.25 ± 0.09 vs. 0.23 ± 0.09, *p* = 0.613). The SUVmax of SAT in the M(1–2) group was significantly lower than that in the M(0) group (0.23 ± 0.09 vs. 0.28 ± 0.08, *p* = 0.002). The SUVmean of the SAT in the MS group was not different from those in the M(0) and M(1–2) groups, whereas the SUVmean of the SAT in the M(1–2) group was lower than that in the M(0) group (0.13 ± 0.05 vs. 0.16 ± 0.06, *p* < 0.001, Fig 4D). Therefore, the SUVmax and SUVmean of SAT differed from those of the VAT, regardless of VAT inflammation.

### Multivariate analysis of the SUVmax and SUVmean of VAT to determine the associated factors in patients with MS

To determine the factors associated with metabolic SUV surrogate markers of VAT in the MS group, we performed multiple linear regression analyses, which included waist circumference and MS components including dyslipidemia, hypertension, and diabetes, and the SUVmax of the spleen and BM as covariates.

In participants with MS not using lipid lowering drug medications, such as statins or fibrate, the SUVmax of the spleen was significantly, positively associated with the SUVmax of VAT (Table 5). Interestingly, anti-hypertensives and anti-diabetic medication were associated with reduced SUVmax of VAT. There was no impact of waist circumference on the SUVmax of VAT, and no association between SUVmean and any of the covariates (Table 5).

In participants with MS not using anti-diabetic medication, the SUVmax of the spleen was positively associated with the SUVmax of VAT (Table 6). Anti-hypertensives and lipid-lowering medications were correlated with decreased SUVmax of VAT. Again, there was no effect of waist circumference on the SUVmax of the VAT. There was also no association between the SUVmean and any of the covariates (Table 6).

**Table 5:** Multiple linear regression analysis using SUV parameters of VAT as dependent variables in MS patients not using lipid lowering drugs (statins or fibrates).

Variable			
Dependent	Independent	*β*	*p*
VAT SUVmax	WC*	-0.003	0.544
	WC^†^	0	0.967
	Triglycerides*	-7.26E-05	0.889
	Triglycerides^†^	0	0.754
	HDL cholesterol*	0.001	0.802
	HDL cholesterol^†^	-0.001	0.905
	Hypertension medication*	-0.384	0.007
	Hypertension medication^†^	-0.313	0.047
	DM medication*	-0.2	0.051
	DM medication^†^	-0.221	0.066
	Spleen SUVmax*	0.155	0.02
	BM SUVmax^†^	0.057	0.495
VAT SUVmean	WC*	-0.001	0.53
	WC^†^	-0.002	0.357
	Triglycerides*	0	0.084
	Triglycerides^†^	0	0.079
	HDL cholesterol*	-0.003	0.321
	HDL cholesterol^†^	-0.002	0.421
	Hypertension medication*	-0.028	0.678
	Hypertension medication^†^	-0.045	0.521
	DM medication*	0.009	0.863
	DM medication^†^	0.012	0.819
	Spleen SUVmax*	-0.036	0.266
	BM SUVmax^†^	-0.016	0.686

MS, Metabolic syndrome; VAT, visceral adipose tissue; SUVmax, maximum standardized uptake value; SUVmean, mean standardized uptake value; WC, waist circumference; HDL, high-density lipoprotein; DM, diabetes mellitus; BM, bone marrow.

*After adjustment of BM for SUVmax, age, gender, body mass index, low-density lipoprotein cholesterol, current smoking status, and high-sensitive C-reactive protein.

^†^After adjustment of spleen for SUVmax, age, gender, body mass index, low-density lipoprotein cholesterol, current smoking status, and high-sensitive C-reactive protein.

**Table 6:** Multiple linear regression analysis using SUV parameters of VAT as dependent variables in MS patients not using DM medications.

Variable			
Dependent	Independent	*β*	*p*
VAT SUVmax	WC*	0.001	0.897
	WC^†^	0.002	0.633
	Glucose*	-0.001	0.749
	Glucose^†^	-0.002	0.576
	Hypertension medication*	-0.251	0.015
	Hypertension medication^†^	-0.197	0.067
	Lipid lowering medication*	-0.248	0.017
	Lipid lowering medication^†^	-0.234	0.036
	Spleen SUVmax*	0.137	0.033
	BM SUVmax^†^	0.064	0.36
VAT SUVmean	WC*	0.002	0.42
	WC^†^	0.001	0.604
	Glucose*	-0.003	0.119
	Glucose^†^	-0.003	0.201
	Hypertension medication*	-0.012	0.821
	Hypertension medication^†^	-0.032	0.556
	Lipid lowering medication*	0.087	0.108
	Lipid lowering medication^†^	0.082	0.151
	Spleen SUVmax*	-0.053	0.117
	BM SUVmax^†^	-0.02	0.591

MS, Metabolic syndrome; VAT, visceral adipose tissue; SUVmax, maximum standardized uptake value; SUVmean, mean standardized uptake value; WC, waist circumference; DM, diabetes mellitus; BM, bone marrow.

*After adjustment of BM SUVmax, age, gender, body mass index, triglycerides, high-density lipoprotein cholesterol, low-density lipoprotein cholesterol, current smoking status, and high-sensitive C-reactive protein.

^†^After adjustment of spleen SUVmax, age, gender, body mass index, triglycerides, high-density lipoprotein cholesterol, low-density lipoprotein cholesterol, current smoking status, and high-sensitive C-reactive protein.

## Discussion

To the best of our knowledge, this is the first study reporting the relationship between VAT inflammation and MS components in human subjects using ^18^F-FDG PET/CT. In this study, we showed that the SUVmax of VAT was significantly higher in patients with MS compared to patients with two or less MS components, and was correlated with systemic inflammation and the number of MS components. However, the SUVmean of VAT was significantly lower in the MS group than that of the M(0) group and was inversely correlated with systemic inflammation.

There is accumulating evidence that systemic inflammation is associated with a variety of local inflammatory conditions, including myocardial infarction, obesity, and periodontal disease, and that this association plays an important role in the development or aggravation of inflammation at distant organs, leading to adverse clinical cardiovascular outcomes [6, 7, 23–26]. Previous study [27] reported that inflammatory activity in atherosclerotic plaques increased with an increased number of MS components. Therefore, local inflammation can affect a distant cardiovascular inflammation via circulating inflammatory mediators and increase the risk of CVD development [2–4]. The inflammatory activity of VAT in visceral obesity can be a significant surrogate maker in CVD risk stratification. Despite this, there is no direct marker for assessing VAT inflammation in clinical practice. To date, VAT inflammation has been evaluated by measuring the amount of general obesity or serological inflammatory markers. However, this does not enable visualization the inflammatory status of VAT in MS patients. Furthermore, several previous studies have been suggested that volumetric measurement of fat depots by CT or magnetic resonance imaging (MRI) is not sufficient to reflect the inflammatory status of VAT which is linked to the increased risk of CVD [8, 28].

Since adipocytes and immune cells constitute the major cell populations in dysfunctional VAT inflammation [11, 29], previous ^18^F-FDG PET/CT studies have reported that the SUVmean of VAT is unexpectedly low in obese people [9, 10] and the SUVmean of the VAT was inversely correlated with visceral obesity [30]. One of previous study [10] suggested that the decreased SUVmean of VAT in obese people might be due to the decreased vascular perfusion of VAT, which is impaired in obesity. Consistent with these results, the present study showed that the SUVmean of VAT was reduced in MS and was inversely associated with systemic inflammatory activity. For the interpretation of this result, it must be considered that dysfunctional adipocytes in inflamed VAT have lower glucose uptake during inflammatory activity, which is associated with the development of insulin resistance in obesity [31, 32]. An increased amount of secreted, non-esterified fatty acids from adipocytes and an increase in adipocyte size, which is associated with a loss of GLUT-4, contribute to the decreased glucose uptake of dysfunctional adipocytes [31, 32]. Activated macrophages can also alter the insulin function in adipocytes via the down-regulation of GLUT-4 in adipose tissue [12, 33]. As VAT is largely composed of adipocytes, the SUVmean of VAT is presumably dependent on the insulin resistance of dysfunctional adipocytes, rather than the inflammatory state of immune cells in VAT. Thus, both the decreased glucose uptake associated with insulin resistance in adipocytes and the decreased vascular perfusion in VAT may contribute to the reduced SUVmean of VAT in MS.

Conversely, during inflammation, glucose uptake is upregulated in macrophages [33, 34], the predominant inflammatory cell type in VAT. Macrophages express GLUT1 for glucose transport in VAT [29] and thus, do not develop insulin resistance due to the absence of GLUT-4, even during inflammation. Therefore, we suspect that SUVmax indicate the maximal inflammatory metabolic activity of immune cells, such as macrophages. This concept is robustly supported by studies involving the use of ^18^F-FDG PET/CT imaging to study atherosclerotic plaques [35, 36]. Consistent with the results of these studies, our findings show that the SUVmax of VAT is elevated in MS and exhibits a significant association with surrogate PET markers of systemic inflammation, such as the ^18^F-FDG SUVmax of spleen and bone marrow. Therefore, this finding using ^18^F-FDG PET/CT in MS is attributed to the different glucose metabolism between adipocytes and immune cells during inflammation.

Anti-diabetic and anti-hypertensive agents have been shown to have anti-inflammatory effects [37, 38]. Several reports indicate that statin and fibrate exert anti-inflammatory effects via the inhibition of macrophage activity, in addition to lipid lowering [39–41]. It is interesting that the present study showed that in MS patients taking anti-diabetic, anti-hypertensive, or lipid lowering drugs, the SUVmax of VAT was lower.

There is a possibility that the high SUVmax is due to delayed and impaired transport of glucose in the vessel endothelium. However, as shown in Table 2, the level of fasting blood glucose showed no significant difference between the comparison groups. Therefore, as ^18^F-FDG is a glucose analogue, we believe that the high SUV max of the VAT could be mainly attributed to the increased glucose metabolism of inflammatory cells such as macrophages.

Interestingly, in addition to glucose uptake of VAT, we also found that the liver SUVmax was significantly higher in MS group than that of M(0) or M(1–2) group (Table 1). Furthermore, liver SUVmax showed significant positive correlation with BMI (*r* = 0.462, *p* < 0.001) and WC(*r* = 0.268, *p* = 0.001), whereas there was no significant correlation with hsCRP. These observations were consistent with previous studies that high liver FDG uptakes were correlated with MS and were determined by BMI and WC [42, 43]. They suggest that elevated liver inflammation in MS may contribute to the increased FDG uptake in liver. However, underlying mechanism is lacking and remains to be determined.

Despite being a prospective study, the present study was conducted as a cross-sectional study in one institute. Therefore, we could not answer the question of whether the inflammatory activity of VAT was a cause or result of MS. Further longitudinal studies to determine whether the reduction of the SUVmax in VAT by therapeutic intervention is subsequently associated with a reduction of CVD risk are warranted. Additionally, we could not control the use of medications (type of drug, dosage, and duration of therapy) for MS that may have affected the inflammatory activity of VAT and obscured the contribution of MS to VAT inflammation. Although ^18^F-FDG PET/CT is a well-known method for the evaluation of VAT glucose uptake, [8–10, 19–21, 30] we were unable to perform histopathological analysis of tissue samples from VAT, which may have added to our findings. ^18^F-FDG PET/CT has some physical limitations on spatial resolution. Thus, cell-specific metabolism can be visualized as clustered radioactive signals which may hinder the precise evaluation of cell-specific metabolism. To minimize this limitation, we selected the same specific retroperitoneal VAT area (Fig 3), which is described in previous study [9], as a representative VAT for image analysis in all participants. Finally, we were not able to control factors that could affect FDG uptake, such as plasma glucose and insulin levels, nor the image acquisition time after tracer injection.

In summary, with increasing number of MS components, the SUVmax of VAT and systemic inflammation increased proportionally, while the SUVmean of VAT decreased. Therefore, the SUVmax and SUVmean of VAT may reflect the unique inflammatory status of VAT, which may contribute to increased CVD risk in MS.
